# Altered expression of pectoral myosin heavy chain isoforms corresponds to migration status in the white-crowned sparrow (*Zonotrichia leucophrys gambelii*)

**DOI:** 10.1098/rsos.160775

**Published:** 2016-11-30

**Authors:** Brandy P. Velten, Kenneth C. Welch, Marilyn Ramenofsky

**Affiliations:** 1 Department of Biological Sciences, University of Toronto Scarborough, 1265 Military Trail, Toronto, Ontario, CanadaM1C 1A4; 2 Center for the Neurobiology of Stress, University of Toronto Scarborough, 1265 Military Trail, Toronto, Ontario, CanadaM1C 1A4; 3 Center for the Analysis of Genome Evolution and Function, University of Toronto, 25 Willcocks Street, Toronto, Ontario, CanadaM5S 3B2; 4 Department of Neurobiology Physiology Behavior, University of California, Davis, One Shields Avenue, Davis, CA 95616, USA

**Keywords:** migration, myosin, muscle

## Abstract

Birds undergo numerous changes as they progress through life-history stages, yet relatively few studies have examined how birds adapt to both the dynamic energetic and mechanical demands associated with such transitions. Myosin heavy chain (MyHC) expression, often linked with muscle fibre type, is strongly correlated with a muscle's mechanical power-generating capability, thus we examined several morphological properties, including MyHC expression of the pectoralis, in a long-distance migrant, the white-crowned sparrow (*Zonotrichia leucophrys gambelii*) throughout the progression from winter, spring departure and arrival on breeding grounds. White-crowned sparrows demonstrated significant phenotypic flexibility throughout the seasonal transition, including changes in prealternate moult status, lipid fuelling, body condition and flight muscle morphology. Pectoral MyHC expression also varied significantly over the course of the study. Wintering birds expressed a single, newly classified adult fast 2 isoform. At spring departure, pectoral isoform expression included two MyHC isoforms: the adult fast 2 isoform along with a smaller proportion of a newly present adult fast 1 isoform. By spring arrival, both adult fast isoforms present at departure remained, yet expression had shifted to a greater relative proportion of the adult fast 1 isoform. Altering pectoral MyHC isoform expression in preparation for and during spring migration may represent an adaptation to modulate muscle mechanical output to support long-distance flight.

## 1. Introduction

Each year, migratory birds travel great distances to take advantage of predictable and ephemeral resources to breed in spring then return to non-breeding sites to survive throughout winter. In response, migrants express a succession of life-history stages each with specific traits of behaviour, physiology and morphology. These alterations are best described as examples of phenotypic flexibility, enabling migrants to synchronize their capacity to respond to the dynamic changes in the energetic demands of each seasonal period [1–3]. For example, migratory birds, such as the white-crowned sparrow (*Zonotrichia leucophrys gambelii*), that winter in temperate regions show fattening to withstand periods of intense weather that may reduce or even eliminate feeding opportunities for prolonged periods [4–6]. Near the conclusion of the wintering stage, birds undergo a prealternate moult that includes replacement of crown and body feathers and some retrices or feathers of the tail [7,8]. This moult constitutes an upgrade of the breeding plumages as well as replacement of feathers for improved insulation and protection from environmental wear and tear experienced throughout the forthcoming migration and breeding [9–11].

In preparation for spring migration and subsequent breeding in the Arctic, these migrants undergo further changes in physiology [12–16], behaviour [17–20] and morphology [21–25] that serve to support the often unpredictable energetic and mechanical demands of long-distance travel, as well as aiding in the adjustment to conditions upon the breeding grounds [2,24,26–28]. Many of these changes include shifts in fat deposition, body mass and conformation [2,24,25,29,30], and differences in the timing and level of flight activity [18,20,31]. In addition, unpredictable abiotic factors encountered throughout this time [32] can directly influence the energetic [14,16] and mechanical [17,33] demands placed on birds and in particular the flight muscles. The variable requirements associated with fuelling and powering flight through periods of seasonal transition present a unique set of challenges that migratory species must meet to sustain optimal flight performance.

Fat or lipids play a significant role in a bird's ability to meet the metabolic demands of sustained, long-distance flight [16,34]. Lipid is the primary fuel of the flight muscle of migratory species as it is more energy rich than either carbohydrate or protein. Also, as lipid within the body is stored without excess water, it contributes marginally to overall weight, a strong consideration for aerial species [12,14,16]. Migratory birds are known to increase lipid stores significantly in preparation for spring migration [14,34–36]. These stores are depleted over the course of the migration due to enhanced capacity for lipid transport to and oxidation within the flight muscles compared with non-migratory periods and species [14,15,37,38]. The seasonally driven changes in extracellular lipid stores and their importance for fuelling sustained flight is well established in many birds [14,16,19,34,38,39]; yet, as a primary site of lipid utilization [37,38], little is known about the role intramuscular lipid stores may play as birds prepare for and complete migration.

In addition to meeting the specific metabolic requirements of migration, the flight muscles must also support the variable mechanical demands associated with long-distance flight. The aerodynamic requirements for flight strongly depend on the body mass that must be supported as birds gain and lose mass throughout the fuelling and flight stages [14,17,35,36,40]. In many avian species, these changes in body mass are accompanied by compensatory changes in flight muscle mass [23–25,29,30], an adaptation often attributed to a species' ability to adjust to variable mechanical demands [17,21,24,29,41]. However, concurrent shifts in body and flight muscle mass are not observed in all long-distance migrants [23]. Further, estimates suggest that hypertrophy of the flight muscle may not be solely adequate to sustain optimal flight performance as body mass increases in preparation for departure of some migratory species [30]. Thus, other biomechanical [33,42,43] and/or behavioural [17,33] mechanisms probably contribute to a species' ability to successfully modulate the power-producing capacity of the flight muscle to maintain optimal performance across life-history stages. Yet, very few studies have examined in detail other potential adaptations for maintaining optimal flight performance as birds progress through the stages from winter, spring departure and arrival on breeding grounds.

Vertebrate sarcomeric myosin is a hexameric protein composed of four light chain and two heavy chain subunits [44]. The myosin heavy chain (MyHC) subunits have been shown to directly correlate with many of the contractile properties of vertebrate muscle fibres [44], including maximum shortening velocity [45–47], force generation [48] and the contractile conditions under which peak power output is generated [49]. Owing to differences in the activity of the myosin adenosine triphosphatase (mATPase) located on the motor domain of the MyHCs [50], these properties can vary significantly among muscle fibres depending on the isoform(s) they express [44–46]. Such differences in MyHC expression produce fibres with specific conditions for optimal contractile performance [49,51]. As the MyHC isoform present within a fibre has the ability to directly influence the mechanical output of a muscle, altering the MyHC expression of avian flight muscles represents a potential adaptation to adjust muscle output in response to the variable mechanical demands experienced by migratory species over the course of a year.

In mammals, histological fibre type generally correlates with MyHC expression, with further subdivisions based on the metabolic properties of the fibre [44]. Studies comparing flight muscle fibre type across avian life-history stages are currently limited [22,24], and fibre-type shifts associated with the migratory status of birds have only been reported for a few species [22], emphasizing the need for more in depth investigations. Nevertheless, the relationship between MyHC expression and histologically defined fibre type is not well established in birds [52], with several examples of similar histologically defined fibre types appearing to contain different MyHC isoforms [53].

Based on this knowledge, we hypothesized that, to meet and fuel the dynamic mechanical requirements associated with long-distance flight, white-crowned sparrows would exhibit a progressive alteration of muscle morphology and fuel stores as they progress from the winter through spring migration stages. Therefore, in this study, we examined alterations in flight muscle fibre biochemistry and protein expression of the migratory Gambel's white-crowned sparrow (*Zonotrichia leucophrys gambelii*) throughout the transitional stages of winter, spring departure and arrival on breeding grounds. In addition to measures of body and muscle mass, we used protein separation techniques and histological fibre typing to identify underlying characteristics of the flight muscle that may be overlooked using fibre typing alone. We found that, although histologically defined fibre type of the sparrow pectoralis did not differ between wintering and migratory stages, intramuscular lipid levels increased as the sparrows prepared for migration and were depleted in arriving birds. Further, pectoral MyHC expression significantly changed as birds prepared for and completed their migrations. To the best of our knowledge, this is the first documented case of pectoral MyHC expression shifting in coincidence with a species' migratory status.

## 2. Material and methods

### 2.1. Collection sites and conditions

Gambel's white-crowned sparrow is a nocturnal, long-distance migrant that travels each spring from wintering grounds that extend from Mexico north to Washington State in the USA to breeding grounds stretching from the US-Canadian border throughout the Arctic [54,55]. Two focal populations of white-crowned sparrows were utilized in this study: wintering flocks residing in fields and hedgerows of agricultural fields in Yolo County on the University of California Davis lands (38°33′ N, 121°44′ W) from early October through mid-April and birds arriving in May at a breeding site north of the Brooks Range of Alaska (68°50'25^″^ N, 148°50'0^″^ W). Birds were trapped at three stages during the first five months of the year. Samples were first collected on 4 February 2014 (*n* = 6, juvenile males) when birds were living in large winter flocks in Yolo County. The next sampling stage was 4 April 2014 (*n* = 1 adult female, 5 adult males) at the same sites in Yolo County. At this time, birds were in small flocks, completing prealternate moult, and had attained adult plumage with black and white crown feathers [7]. Records confirm that 4 April is approximately 12 days prior to the mean annual departure date for white-crowned sparrows from Yolo County [56] and represents a preparatory period for spring departure. Final samples were collected on 12 and 14 May 2014 (*n* = 9 adult males), within 3 days of the first sightings of white-crowned sparrows along the Haul Road of the Trans Alaskan Highway located within 1 km SW of Pump Station 3 (S. Beaudreault and M.R. 2014, personal observations). At this time, birds were found in large flocks in Alaskan Willow (S*alix alaxensis)* breaks, approximately 75 m from the road and observed feeding on seeds and insects in exposed bare patches amidst the snow and ice cover.

At all sites, birds were captured in mist nets or potter traps baited with commercial bird seed. Immediately upon capture, birds were weighed to the nearest 0.1 g using a Pesola spring balance. Body size was assessed with length measurements of the relaxed wing, tarsus and beak using calipers (to the nearest 0.1 mm). Next, fat score was assessed visually by observing the presence of lipid in the coelomic cavity and chorico-clavicular fossa with values that ranged from 0, no observable fat, to 5, bulging fat deposits [1,57]. Both scores were summed for statistical analyses. Flight muscle size was visually assessed with a scoring system for muscle profile that is based on four classes ranging from 0 to 3 [57–60]. Specifically, a score of 0 presents a sharp protruding edge of the keel to the touch and an extreme concave muscle mass, typical of emaciated birds. A score of 1 still has a prominent edge of the keel but the muscle is beginning to bulge out slightly. For a 2, the muscle mass is convex in shape but the edge of the keel edge remains evident to the touch, and for a 3 the pectoralis rises above the keel that is now embedded in the bulging muscle.

Prealternate body moult was scored on three body regions: crown, back (including nape, back and rump) and abdomen (including throat, breast, abdomen and flanks). Each region was given a score between 0 and 3 depending on the extent of moulting feathers: 0, no moult; 1, light moult (1–15% area); 2, moderate moult (16–50% area) and 3, heavy moult (51–100% area). Scores of each region were summed to generate a total moult score for each sampling period [57].

Next, birds were quickly transferred to the laboratory where they were deeply anaesthetized with 5% isoflurane (Baxter Laboratories, Ill.) and euthanized by decapitation. Flight muscle samples (including both the pectoralis and supracoracoideus) were excised bilaterally and weighed to the nearest 0.001 g on an electronic balance. Excised muscles were then wrapped in aluminium foil and initially placed on dry ice before being stored at −80°C until transportation to the University of Toronto for analysis. All procedures and animal handling were done in accordance with University of California Davis Institutional Animal Care and Use Committee (IACUC—Protocol no. 17144).

### 2.2. Histology

Blocks of tissue measuring approximately 1 cm wide by 4 cm long were cut through the entire depth of the midbelly of the sternobrachialis portion of the pectoralis. Blocks were coated in Tissue-Plus O.C.T. Compound (Fisher Healthcare) and frozen in isopentane cooled by liquid nitrogen. Serial transverse sections approximately 12 µm thick were cut in a cryostat (Leica CM3050 S, Leica Biosystems) operating at −20°C and mounted on glass slides (Superfrost Plus, Fisher Scientific). Slides were stored at −20°C until staining occurred.

Histological fibre type was based on both myosin expression and metabolic properties. Serial sections were stained for mATPase activity under both acidic (pH = 4.25) and basic (pH = 10.4) pre-incubation conditions [61,62], succinate dehydrogenase (SDH) activity [61,63], and the presence of lipid using oil red O (ORO) [64]. Fibres stained for mATPase were classified as either fast-twitch or slow fibres [61]. SDH-stained sections were classified as oxidative or glycolytic based on their relative staining intensity [65]. Lipid density was categorized as high (deep red) or low (pale pink) using ORO-stained sections. The gastrocnemius of the ruby-throated hummingbird (*Archilochus colubris*), known to contain both fast-twitch and slow fibres [66], was used as a control in mATPase staining, while the pale staining, glycolytic fibres of chicken (*Gallus gallus*) superficial pectoralis [61] were used as a control for SDH-stained sections.

Stained slides were viewed using a Zeiss Epifluorescence microscope in brightfield mode at 200× magnification. Randomly selected sections were digitized using an AxioCam High Resolution colour camera. Fibre type by count was determined by counting all whole fibres that fell within the 1300 × 1030 pixel image area. Fibre counts were only performed using mATPase and SDH-stained sections. For each individual, at least 500 total fibres were counted for each stain. The diffusive nature of ORO-stained sections did not allow individual fibres to be reliably identified for counting. As ORO-staining intensity visually appeared to correlate with the relative coloration of SDH-stained sections, we tested if SDH-staining may serve as a proxy for lipid density by comparing the relative proportion of high/low lipid density regions within the muscle section to the specific coloration of serial SDH-stained sections. The number of pixels composing high or low lipid regions of ORO-stained sections and blue- and purple-hued regions in SDH-stained sections was determined by digitally tracing these regions in at least nine serial sections of both wintering and arriving sparrows using ImageJ (v. 1.49). While use of serial sections enables us to directly compare between ORO- and SDH-stained sections to determine if relative staining of the two techniques is correlated, these sections were specifically chosen based on the presence of recognizable ‘landmarks’ (i.e. distinct patterns of fascia, the presence of blood vessels) and for a diversity of fibre staining, and thus, may not necessarily reflect the average nature of staining for each specimen across the entire section.

### 2.3. SDS-PAGE electrophoresis and western blots

Sample and gel preparation, silver staining and relative densitometry measurements were carried out as in Velten & Welch [52]. MyHC gels were run between 4 and 9°C for approximately 45 h. For all specimens expressing multiple MyHC isoforms, samples were run in triplicate on separate gels. Samples prepared from the superficial pectoralis, anterior latissimus dorsi (ALD) and lateral gastrocnemius of the domestic chicken were used as controls and to aid in the identification of sparrow MyHC isoforms [67].

A separate set of SDS-PAGE gels were used for western blots to confirm identification and aid in characterization of isoform bands. Gels were transferred to nitrocellulose membranes at 100 V for 2 h in Towbin buffer (25 mM Tris, 192 mM glycine, 20% methanol, 0.1% SDS) with 800 µl of β-mercaptoethanol added to each litre of buffer. Six commercially available, monoclonal antibodies known to react with fast or slow chicken MyHC isoforms were obtained from the Developmental Studies Hybridoma Bank (table 1) [53,66]. Membranes were blocked for an hour at room temperature in blocking buffer (5% skim milk in Tris-buffered phosphate with 0.1% Tween-20). Primary antibodies were diluted in blocking buffer to 0.2–0.4 μg ml^−1^. Both primary and secondary antibody (goat anti-mouse IgG, Sigma Aldrich) incubations occurred at room temperature for 1 h. Blots were developed using the BioRad Clarity Western ECL Substrate and imaged with a BioRad ChemiDoc XRS+.

**Table 1.** Avian myosin heavy chain (MyHC) specificity of the antibodies used in this study (Rosser *et al.* [53]; Welch & Altschuler [66]).

antibody name	myosin heavy chain specificity	dilution (μg ml^−1^)
F30	all fast isoforms	0.4
AB8	adult fast isoform	0.2
EB165	adult + embryonic 1, 2 and 3 fast isoforms	0.2
2E9	neonatal fast isoform	0.4
B103	embryonic 1 and 3 and neonatal fast isoforms	0.2
NA8	slow myosin 2 isoform	0.2

### 2.4. Mass spectrometry

To further characterize the pectoral isoforms of the white-crowned sparrow, MyHC bands from a separate SDS-PAGE gel were used for mass spectrometry. The pectoral isoform of the chicken was used as a control. Bands were stained with Coomassie Brilliant Blue and excised from the gel for trypsin digestion. Samples were analysed on a linear ion trap-Orbitrap hybrid analyzer (Thermo LTQ-XL-Orbitrap Hybrid Mass Spectrophotometer) outfitted with a nanospray source and EASY-nLC 1200 nano-LC system. The instrument method consisted of one MS full scan (400–1400 *m*/*z*) in the Orbitrap mass analyzer, an automatic gain control target of 500 000 with a maximum ion injection of 500 ms, one microscan and a resolution of 30 000. Six data-dependent MS/MS scans were performed in the linear ion trap using the three most intense ions at 35% normalized collision energy. The MS and MS/MS scans were obtained in parallel fashion. In MS/MS mode automatic gain control targets were 10 000 with a maximum ion injection time of 100 ms. A minimum ion intensity of 1000 was required to trigger an MS/MS spectrum. The dynamic exclusion was applied using an exclusion duration of 145 s.

As myosin protein sequences are not currently available for the white-crowned sparrow, proteins were identified by searching MS/MS spectra against the protein sequence database for the chicken, along with a database compiled of newly available, predicted protein sequences extracted from NCBI for the closely related white-throated sparrow (*Zonotrichia albicollis*) using the Thermo Scientific Proteome Discoverer (v. 2.0.0.802). A fragment ion mass tolerance of 0.8 Da and a parent ion tolerance of 30 ppm were used. Up to two missed tryptic cleavages were allowed. Methionine oxidation (+15.99492 Da), cysteine carbamidomethylation (+57.021465 Da) were set as variable modifications.

### 2.5. Statistics

Initially, the distributions of all variables were tested for normality with the Shapiro–Wilk test. Given that the Alaskan birds may represent a different population of white-crowned sparrows than those captured in Yolo County, we tested for population differences in body size using linear measures of wing and tarsus lengths. As scaling relationships across different populations could differ, we used scaled condition indices based on wing length for comparisons of body mass, henceforth referred to as body condition and flight muscle mass [68]. Comparisons across the three stages were accomplished with an analysis of variance (ANOVA) followed by post hoc testing with Tukey's HSD. Fat and moult scores were not normally distributed; therefore, Kruskal–Wallis tests were used to compare across the three stages or Mann–Whitney *U*-tests for independent comparisons. The categorical variable of muscle profile was analysed using a generalized linear model with a multinomial probability function. Mann–Whitney *U*-tests were run for these post hoc analyses with Bonferroni corrections. For the relative proportion of each MyHC isoform, a Kruskal–Wallis test was used to determine if there was significant variation across the three sampling periods. If the variation was significant, a post hoc Dunn's test with Bonferroni correction was performed. Per cent fibre type data were logit transformed using a 0.01 correction factor added to both the numerator and denominator of the transformation equation [69]. Correlation between lipid density and SDH-staining coloration was determined using a linear regression. An ANOVA was used to compare wing and tarsus length, body condition, flight muscle mass and per cent fibre type across the three stages. When significant variation was found, a post hoc Tukey's HSD test was performed. Results were considered statistically significant if *p* < 0.05. Data for body condition, muscle mass, fibre type and relative proportion of MyHC are reported as mean ± s.d. All analyses were performed using R (v. 3.2.1) or SPSS Statistics Software (v. 22.0, IBM, Chicago).

## 3. Results

### 3.1. Morphometrics, body condition and flight muscle

Both tarsus and wing lengths were found to be normally distributed (wing length, *W* = 0.95, *p* = 0.31; tarsus length, *W* = 0.92, *p* = 0.07) and neither measurement revealed significance differences across age (wing: *F*_1,20_ = 1.02, *p* = 0.32; tarsus: F_1,20 _= 0.35, *p* = 0.5), sex (wing: *F*_1,20 _= 3.02, *p* = 0.098; tarsus: *F*_1,20 _= 1.022 = 1.49, *p* = 0.24), or stage (wing: *F*_2,20_ = 0.72, *p* = 0.50; tarsus: *F*_1,20 _= 0.16, *p* = 0.85). Therefore, measurements for age and sex were combined.

**Figure 1.**
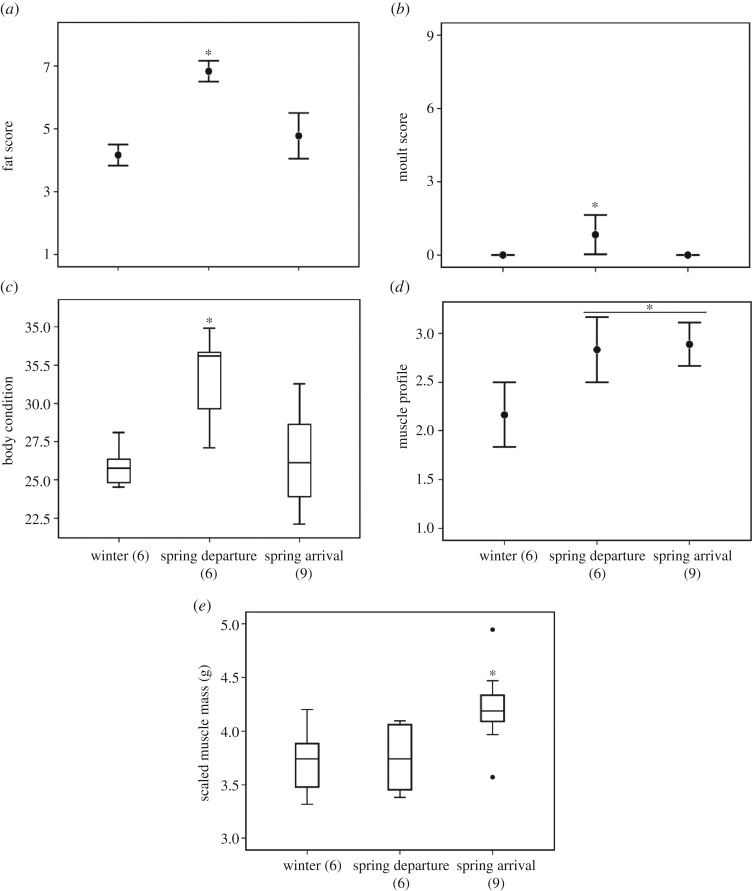
Fat (*a*) and prealternate moult (*b*) scores, body condition (*c*), scaled muscle mass (*d*) and muscle profile score (*e*) for white-crowned sparrows sampled during the winter, and the departing and arriving stages of their spring migration. Asterisks denote statistical difference between stages (*p* < 0.05). Numbers in parentheses denote sample size.

Fat scores at spring departure exceeded those of either winter or arrival (*χ*^2 ^= 13.22, d.f. = 2, *p* = 0.001) (figure 1*a*). Prealternate moult was detected only during spring departure (*χ*^2^ = 8.3, *p* = 0.02) (figure 1*b*). Significant variation was found across the stages for body condition (ANOVA, *F*_2,20_ = 9.89, *p* = 0.001) (figure 1*c*) and muscle profile varied over the course of the study (*χ*^2^ = 6.82, d.f. = 2, *p* = 0.03) with spring departure and arrival exceeding the wintering stage (*U* = 8.5, *p* = 0. 002) (figure 1*d*). Furthermore, scaled flight muscle mass was greatest at spring arrival (*F*_2,20_ = 5.04, *p* = 0.02) compared with specimens collected during winter (*p* = 0.037) and departing (*p* = 0.045) stages (figure 1*e*).

### 3.2. Fibre type and cross-sectional area

**Figure 2.**
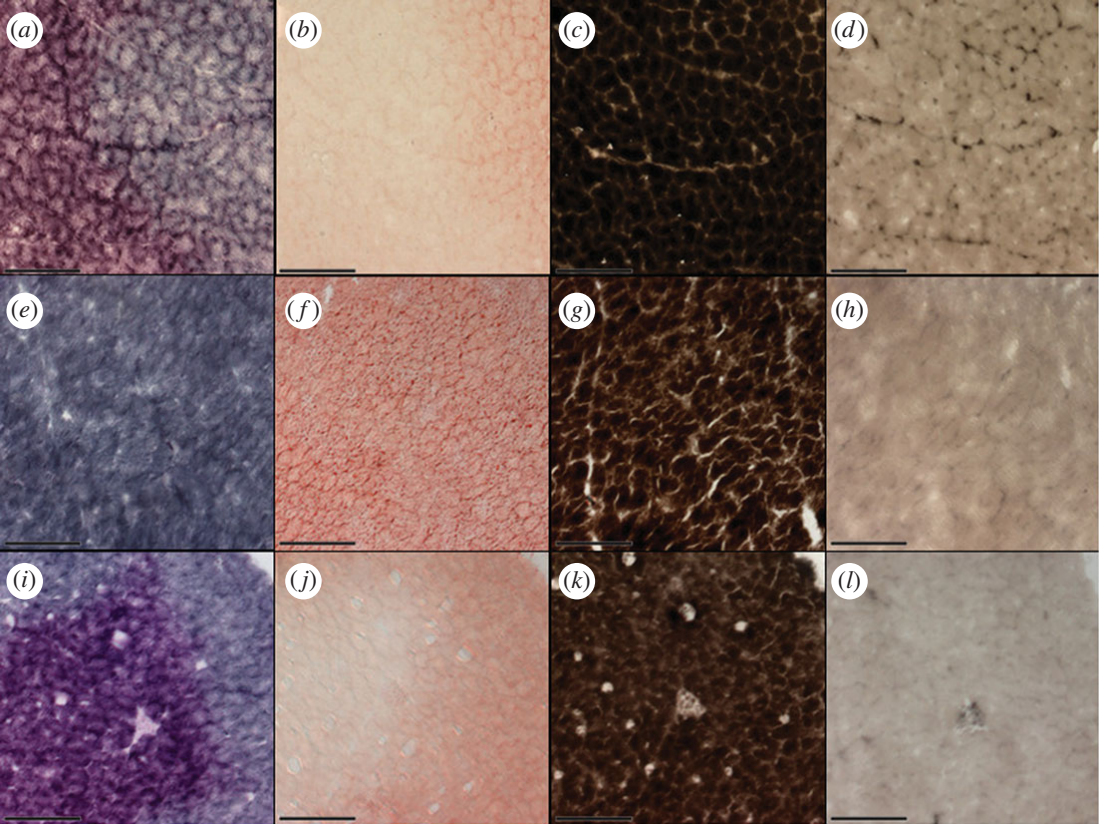
Pectoral serial sections from wintering (*a*–*d*), departing (*e*–*h*) and arriving (*i*–*l*) white-crowned sparrows stained for succinate dehydrogenase (*a*,*e*,*i*), oil red O (*b*,*f*,*j*), and myosin adenosine triphosphatase following basic (*c*,*g*,*k*) and acidic (*d*,*h*,*l*) pre-incubation. Scale bars, 100 µm.

At all three stages, the pectoralis was composed entirely of fast-twitch fibres, as indicated in mATPase-stained sections by the dark staining of basic pre-incubated sections and light staining of sections pre-incubated in acidic medium (figure 2*c*,*d*,*g*,*h*,*k*,*l*).

Although only a single fibre type was identified using the mATPase staining protocol, differences in staining were observed in SDH-stained sections. In wintering and arriving individuals, there was a distinguishable difference in the coloration of fibres, with some fibres appearing blue while others stained with a deep purple hue. When compared with serial ORO-stained sections, the differences in SDH coloration appeared to generally correspond to the relative presence of lipid in the section (figure 2*a*,*b*,*i*,*j*), such that blue-staining fibres generally appeared to have a higher density of lipid (darker red in ORO-stained sections), while those that were more purple in colour tended to have less lipid (no colour/pale pink in ORO-stained sections). The relative pixel area of high and low lipid density regions in ORO-stained sections showed a significant positive correlation to blue and purple coloration of SDH-stained sections, respectively (*R* = 0.4821, *p* = 0.036).

When fibres were classified by colour (blue versus purple), both wintering and arriving sparrows exhibited predominately purple fibres (table 2) with correspondingly pale ORO-staining intensity. Muscle sections from departing birds exhibited a relatively homogeneous dark red ORO-staining, and accordingly, almost all SDH-stained fibres were blue in coloration, with only two birds of the six collected containing any discernable purple fibres (figure 2*e*,*f* and table 2). The relative proportions of blue and purple fibres exhibited significant variation across the three stages (ANOVA, *F*_2,?_ = 30.72, *p* < 0.001), with the muscle of departing birds having significantly fewer purple fibres than that of wintering (*p* < 0.01) and arriving (*p* < 0.01) sparrows. The pectoralis of both wintering and arriving birds did not differ significantly in the relative proportion of blue- or purple-hued fibres (*p* = 0.92).

**Table 2.** Relative fibre type of the pectoralis of white-crowned sparrows across three different life stages: wintering, prior to spring departure and arrival at spring breeding grounds.

	relative fibre type by count				
	myosin ATPase		succinate dehydrogenase		
			% oxidative		
life stage	% fast-twitch	% slow-twitch	purple coloration	blue coloration	% glycolytic
winter	100	0	88.7 ± 9.9	11.3 ± 9.9	0
spring departure	100	0	5.6 ± 13.3	94.4 ± 13.3	0
spring arrival	100	0	81.0 ± 17.1	19.0 ± 17.1	0

Although differences in coloration were observed, the overall intensity of SDH-staining, previously used to classify subtypes of fast-twitch fibres in avian species [61,70], did not vary in a distinguishable manner between blue and purple fibres (electronic supplementary material, figure S1) For the purpose of this study, both fibres were classified as highly oxidative due to their relatively dark staining intensity. Thus, the pectoralis of white-crowned sparrows was composed entirely of fast-twitch, highly oxidative (FOG) fibres, and this fibre composition did not differ across the three stages, although intrafibrillar lipid density did appear to differ across life stages.

### 3.3. Myosin heavy chain expression

**Figure 3.**
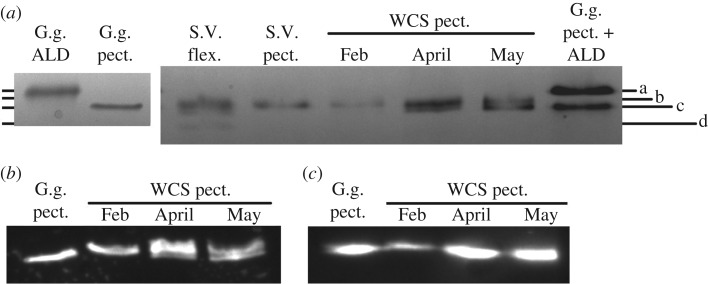
Pectoral myosin heavy chain (MyHC) expression of white-crowned sparrows (WCS) during the winter (February) and the departure (April) and arrival (May) stages of their spring migration. Silver-stained SDS-PAGE gels (*a*) demonstrated that MyHC isoforms transitioned in the sparrow across the three sampling periods. Wintering specimens (February) expressed a single MyHC isoform similar to a novel isoform found in the yellow-bellied sapsucker (S.V.) flexor perforans digiti II (flex.) and pectoralis (pect.) [52]. This isoform migrated between the SM2 (*a*) and adult fast (*c*) isoforms present in the chicken (G.g.) anterior latissimus dorsi (ALD) and pectoralis (pect.), respectively. Departing (April) and arriving (May) sparrows expressed two MyHC isoforms including the novel isoform (*b*) and the adult fast isoform (*c*). No embryonic/neonatal MyHC isoforms, including the isoform present in the woodpecker flexor (*d*), were present in the white-crowned sparrow flight muscle. Both sparrow MyHC isoforms reacted with the EB165 (*b*) and F30 (*c*) antibodies, similar to the adult fast MyHC found in the chicken pectoralis.

Wintering birds expressed a single pectoral MyHC isoform (table 3). On gels, this isoform migrated between the adult fast band of the chicken pectoralis and the slow myosin (SM) 2 band of the chicken ALD (figure 3*a*). Currently, no avian MyHC isoform with a recognized classification (e.g. slow versus fast, adult versus embryonic) is reported as migrating to this gel position. Departing sparrows exhibited different pectoral MyHC expression than wintering birds. The pectoralis of these specimens contained two MyHC isoforms, a darker band that migrated at an identical location as the uncharacterized isoform seen in wintering sparrows, as well as a relatively faster migrating band that migrated to a similar gel position as the adult fast isoform of the chicken pectoralis (figure 3*a* and table 3). Most birds arriving at the spring breeding grounds exhibited the same two pectoral isoforms observed in departing birds (figure 3*a*); however, the expression differed such that the band corresponding to the adult fast isoform had relatively greater expression (table 3). Two of the nine arriving specimens sampled at the breeding grounds expressed only the band corresponding to the adult fast isoform, leading to greater overall variation in this sample group. There was a significant difference in the relative proportion of the two pectoral adult fast isoforms across each of the three sampling periods (wintering versus departing *p* = 0.009, departing versus arriving *p* = 0.008 and wintering versus arriving *p* < 0.001).

**Table 3.** Myosin heavy chain (MyHC) expression in the pectoralis of the white-crowned sparrow at three different life stages: wintering, prior to spring departure from wintering grounds, and spring arrival at breeding grounds.

	per cent composition (mean ± s.d.)		
	life stage		
pectoral MyHC isoform	winter (%)	spring departure (%)	spring arrival (%)
adult fast 1	0 ± 0	35.2 ± 8.9	60.6 ± 24.7
adult fast 2	100 ± 0	64.8 ± 8.9	39.4 ± 24.7

The single isoform present in the wintering stage specimens, along with both isoforms in departing and arriving birds reacted with the EB165 (figure 3*b*) and F30 (figure 3*c*) antibodies. Neither of these bands showed any reactivity with the AB8 antibody, previously shown to react only with the Galliform adult fast isoform [53], any of the antibodies specific for only embryonic and/or neonatal fast isoforms, or the SM 2 antibody (electronic supplementary material, figure S2). This antibody specificity, which was similar to that of the adult fast isoform present in the chicken pectoralis, along with the histological fibre type observed for the sparrow flight muscle in this study, suggests that the unclassified MyHC is a fast avian isoform that contains similar epitopes to the classically identified adult fast isoform of the chicken. None of the commercially available chicken MyHC antibodies were able to discern between the two bands in the white-crowned sparrow pectoralis.

Mass spectrometry confirmed that the two MyHC bands of the white-crowned sparrow pectoralis corresponded to separate protein isoforms of myosin, based on a comparison with the predicted protein sequences for the congener species, the white-throated sparrow (table 4). When compared only to the protein sequence database of the chicken, both pectoral bands of the white-crowned sparrow had the highest consensus with the chicken adult fast MyHC (myosin, heavy chain 1E, skeletal muscle (*Gallus gallus*); GenBank accession no. 61657939). The white-throated sparrow genome is not currently annotated to a degree that enables us to correlate the predicted identity of the white-crowned sparrow myosin isoforms with the classically used terminology of avian MyHCs (i.e. adult fast, embryonic fast 1, etc.). However, based on the antibody reactivity and MS/MS spectra of these two isoforms, we have termed the slower migrating of the two bands a second adult fast isoform (adult fast 2), while the lower band that migrates with the chicken adult fast isoform has been classified as the adult fast 1 isoform.

**Table 4.** Protein identification of white-crowned sparrow pectoral myosin heavy chain (MyHC) isoforms based on comparison of peptide sequences obtained from tandem mass spectrometry with predicted protein sequences from the congener white-throated sparrow (*Zonotrichia albicollis*).

estimated mol. mass (kDa)	identified *Zonotrichia albicollis* protein	GenBank accession no.	apparent mol. mass (kDa)	no. sequenced peptides	coverage (%)	MyHC isoform classification
189.3	PREDICTED: myosin heavy chain, skeletal muscle, adult-like	929512244	190.7	129	56.92	adult fast 2
187	PREDICTED: myosin-4-like	929512236	187.7	119	62.01	adult fast 1

## 4. Discussion

As attributed to many long-distance migrants, Gambel's white-crowned sparrows demonstrate remarkable phenotypic flexibility in terms of behaviour [18,20], physiology and morphology as they transition across life stages throughout the year. Our results offer support of our hypothesis and illustrate significant changes in prealternate moult, fuelling, body condition and flight muscle morphology as birds progress from winter, spring departure and arrival on breeding grounds. Sampling white-crowned sparrows at these three time points provides a timely window into the development and expression of several migratory traits as birds prepare for and execute long-distance flight. In contrast with winter, birds sampled near the time of departure had nearly completed moult with a maximal moult score of 2. Higher scores with a maximum of 9 are observed in March in both field and laboratory studies indicating heavy moult [57]. At departure, with the energetically costly moult near completion, birds were well fuelled with elevated fat levels that contributed to the observed increase in body condition. ORO- and SDH-stained sections of the pectoralis also indicated a relatively uniform, high density of lipid in departing birds compared with wintering or arriving specimens. Upon arrival on the breeding grounds, the histological staining techniques and lipid profiles both demonstrated a decrease in muscle and fat stores at the end of the migratory journey. Thus, the three sampling periods utilized in this study provided distinct physiological parameters for comparative analyses.

To the best of our knowledge, this is the first study to document an altered expression of MyHC isoforms in the pectoralis muscle as birds transition from the wintering to the migratory stage in the spring. As white-crowned sparrows prepare to depart for spring migration, the pectoral MyHC expression changed from a single MyHC isoform, classified as the adult fast 2 isoform in wintering specimens, to two different MyHC isoforms: the adult fast 1 and 2 isoforms. Furthermore, the two MyHC isoforms present in departing birds largely persisted through the course of migration but the relative proportion of the MyHC isoforms upon arriving on the breeding grounds showed a continued progression towards a greater proportion of the adult fast 1 isoform compared with departing individuals.

In contrast with MyHC expression, pectoralis histological fibre type did not differ across the three stages, with the muscle containing only a single highly oxidative, fast-twitch fibre type. Unlike mammals [44], the histological methods used to classify avian fibre types did not discern a difference in fibre type as MyHC expression changed. Thus, the histologically defined fibre type of the white-crowned sparrow pectoralis did not correlate with the specific MyHC(s) expressed by the fibre. While similar uniformity in flight muscle fibre type between wintering and migratory stages has previously been reported in other avian migrants [22], the results of this study demonstrate that a change in MyHC expression may occur throughout the spring migration substages within populations of avian fibres with a consistent histological fibre type.

In mammals, shifts in myosin expression have been documented following artificial manipulation of muscle stimulation patterns and mechanical load, as well as being well documented in cases of disease and ageing [71]. However, changes in fibre type/MyHC isoform expression in healthy mammalian muscle under more natural physiological conditions appear to be a relatively rare, and only apparent following specific resistance training regimes [71–73]. In other vertebrates, the MyHC isoforms of the swimming muscle of some fish species have been shown to change following acclimation to different water temperatures, enabling individuals to maintain swimming performance [74]. In white-crowned sparrows, however, the initial alteration of pectoral MyHC expression occurred on the wintering grounds before the onset of migratory flight, suggesting that the original shift in myosin expression was not the result of a ‘training effect’ due to enhanced muscle use.

Similarly, flight muscle hypertrophy in captive red knots (*Calidris canutus*) has been observed before commencement of either nocturnal flight activity associated with migration or general increased activity. In this case, the authors hypothesized that this change in muscle morphology may be regulated by an endogenous circannual rhythm [75]. As with red knots, white-crowned sparrows exhibited greater muscle profile during preparation for spring departure but before the appearance of nocturnal migratory restlessness and without any indication of increased daytime locomotor activity [20,57]. However, flight muscle hypertrophy and lipid deposition have been observed to occur in concert with increased flapping activity in other migratory species, such as eared grebes (*Podiceps nigricollis*) at the conclusion of the spring stopover en route to breeding grounds [25]. Thus, for some migratory species, pre-departure flight muscle hypertrophy, along with the initial shift in pectoral MyHC expression observed in this study, manifests despite a lack of change in flight or locomotor activity (i.e. training effect). Such finding suggests that these morphological changes may be controlled by a seasonally regulated mechanism similar to other preparatory processes, specifically those associated with the seasonal increase in photoperiod that occurs prior to spring migration [38,39,76]. Such distinctions across migratory species are of interest and deserve further investigation.

The timing of this shift in white-crowned sparrows also differs from that observed in migratory salmon smoults (*Salmo salar*), where changes in myosin expression were observed in the swimming muscle only at the end of the downstream migration in the autumn, following periods of active swimming [77]. This distinction may suggest a difference in the timing mechanisms and possible requirements for the impending movement depending on species and/or type of locomotion, flight or swimming.

Concurrent with the primary change in pectoral MyHC expression, sparrows also exhibited increased body condition as they prepared to depart on their spring migration. This rise in body mass increases the demand for aerodynamic power required to sustain flight at optimal airspeed [17,30,78]. To ensure rapid and efficient flight speeds over the course of a migration, the capacity of the flight muscle to generate mechanical power must be sufficient to meet the aerodynamic requirements [17,79]. In many migratory species, significant hypertrophy of the flight muscle prior to migration is attributed to enabling birds to meet the enhanced demand for mechanical power at departure when birds are at their heaviest [12,21,24,30,31,75]. Although flight muscle profiles of departing sparrows appeared large and protruded beyond the keel, indicating enlarged flight muscles, muscle mass of white-crowned sparrows increased only slightly between the wintering and departing stages. This observed change in muscle profile may be attributed, in part, to the increased level of intramuscular lipid observed in departing sparrows (this study and [80]). Thus, while flight muscle hypertrophy in white-crowned sparrows prior to departure is apparent via measures of muscle profile, it did not manifest as a significant change in muscle mass, as has been reported in other migratory species [21,24,25,30,36,75] but, rather, appeared upon arrival, suggesting a contribution of flight itself. The timing of muscle mass increase may be attributed to a power training effect of migratory flight that would enhance muscle hypertrophy, electrolyte and water balances, all contributing to increased muscle mass [81].

As the contractile properties of a muscle strongly correlate with MyHC expression [44,46,48], a shift in the MyHC isoform(s) present can alter the inherent mechanical properties of the muscle. Given the timing of the initial observation of altered pectoral myosin expression in departing white-crowned sparrows, this shift may represent an adaptation to modulate the mechanical power-generating capacity of the flight muscle to meet the variable mechanical demands associated with departure and sustained flight. Such a shift, without a substantial increase in muscle mass, may permit white-crowned sparrows to enhance the power-generating capacity of the flight muscle while bypassing potential constraints to significantly increasing muscle size [30]. These constraints include the ability to rapidly and efficiently deliver oxygen and fuel to active muscle, as well as the increased metabolic cost of flight due to the effect of enlargement of this tissue on overall body mass and the maintenance costs of hypertrophied muscle.

Over the course of the migration, flight muscle mass continued to increase as pectoral MyHC expression of the white-crowned sparrow shifted towards a greater relative proportion of the adult fast 1 isoform. The trend towards greater flight muscle mass upon arrival to the breeding grounds has been repeatedly observed in white-crowned sparrows over multiple migratory seasons (M.R. 2014, personal observation). However, this finding contrasts with other long-distance migrants, many of which exhibit decreased flight muscle mass following migration due to breakdown of the muscle for use as a protein fuel source [14,16,35,82]. Some of these cases suggested that birds may arrive in poor condition. However, white-crowned sparrows are long-distance, short-bout migrants that do not cross immense ecological barriers. Thus, the birds have access to food and water at stopover sites en route. Unless confronted by intense storms or conditions that impede feeding or access to critical resources, it is unlikely that they will catabolize flight muscle as a protein fuel reserve to any great extent during their migration. Rather, white-crowned sparrows appear to exhibit an exercise-induced increase in flight muscle size over the course of spring migration [73]. Currently, it is unclear whether the trend towards a higher proportion of the adult fast 1 isoform in arriving sparrows may also be related to training effects over the duration of the flight or reflects a continuation of the process initiated prior to departure.

Currently, measurements of the contractile properties of fibres expressing different avian MyHC isoforms are restricted to those present in the muscles of the domestic chicken [45,46]. Therefore, it is difficult to know how the properties of the adult fast 2 isoform may compare to those of the adult fast 1 MyHC or how this transition may affect the mechanical output of the pectoralis. Further research into the functional differences of the various MyHC isoforms in the white-crowned sparrow pectoralis would help to identify the adaptive significance behind the observed shift, such as changing mechanical needs during flight, as well as other potential drivers including muscle efficiency [83], and/or changing thermoregulatory demands [84].

In conclusion, white-crowned sparrows significantly altered moult status, fat deposition, body condition as well as size and morphology of their flight muscle as they progressed from wintering through the spring migratory stage. The observed shift in MyHC isoforms present in the pectoralis appears to be the first documentation of a change in the MyHC expression of avian flight muscle coinciding with migratory status. The shift in the morphological characteristics of the flight muscle occurred over a period when white-crowned sparrows experience significant morphological, behavioural, and environmental changes as they prepare for and complete spring migration. During this time, the mechanical demands placed on the muscle probably vary due to factors including changes in body size and unpredictable environmental conditions, requiring sparrows to modulate the output of their flight muscle to maintain optimal performance [17,79]. This study suggests that white-crowned sparrows adjust both the size and MyHC isoform expression of their flight muscle---adaptations that may confer benefits both to birds en route and upon arrival at the breeding grounds when faced with unpredictable conditions in the arctic [28]. Furthermore, the shift in MyHC expression was not associated with a coincident change in histologically defined fibre type in the pectoralis, suggesting the possibility that classic histological methods may not be sufficient or sensitive enough to account for the diversity (or lack thereof) of myosin expression in avian muscle [52].

## Supplementary Material

Supplementary Figure 1: Comparison of RGB and greyscale images of SDH-stained sections

## Supplementary Material

Supplemental Figure 2. Reactivity of pectoral (pect.) MyHC isoforms from wintering (2/4/14), departing (4/14/14), and arriving (5/14/14) white-crowned sparrows with the B103 (A), E29 (B), AB8 (C), and NA8 (D) antibodies

## Data accessibility

Supporting data for this research can be accessed online in the Dryad Data Repository: http://dx.doi.org/10.5061/dryad.t92g5 [85].
