# Supplementary figures and images for: Altered expression of pectoral myosin heavy chain isoforms corresponds to migration status in the white-crowned sparrow (Zonotrichia leucophrys gambelii)
Source: R Soc Open Sci. 2016 Nov 30;3(11):160775. doi: 10.1098/rsos.160775 (PMC5180162; doi:10.1098/rsos.160775)

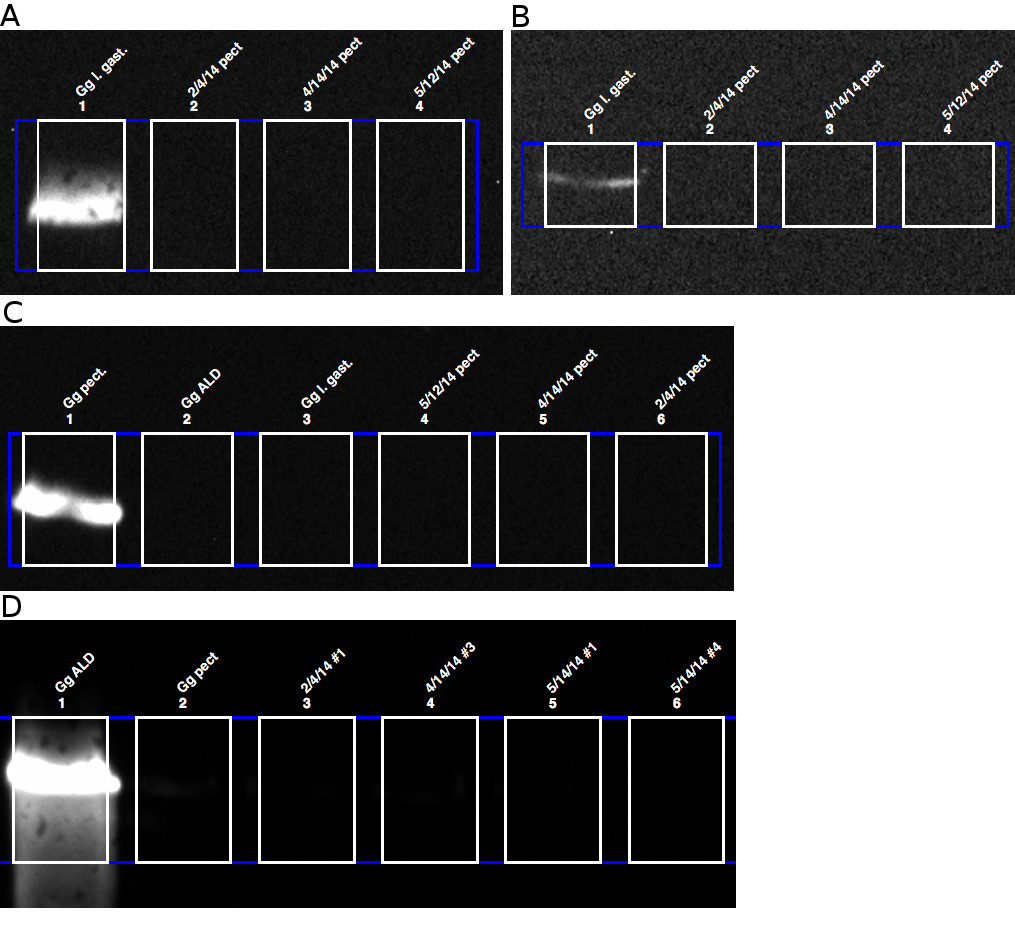

Supplement: Supplementary Figure 1: Comparison of RGB and greyscale images of SDH-stained sections [file rsos160775supp1.jpg]

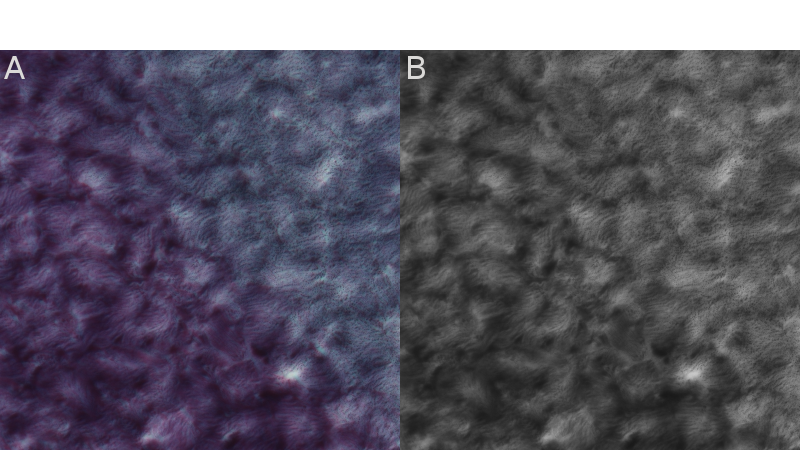

Supplement: Supplemental Figure 2. Reactivity of pectoral (pect.) MyHC isoforms from wintering (2/4/14), departing (4/14/14), and arriving (5/14/14) white-crowned sparrows with the B103 (A), E29 (B), AB8 (C), and NA8 (D) antibodies [file rsos160775supp2.png]
